# Effect of Caffeine on Cell Death, Oxidative Stress, and Microglial Morphology in a Ferret Organotypic Brain Slice Model of Hypoxia–Ischemia

**DOI:** 10.3390/neurosci7040079

**Published:** 2026-07-10

**Authors:** Olivia C. Brandon, Kylie A. Corry, Zheyu Ruby Jin, Kate F. DiNucci, Matthew J. Magoon, Nels Schimek, Daniel H. Moralejo, Sandra E. Juul, Patrick M. Boyle, Elizabeth A. Nance, Thomas R. Wood, Sarah E. Kolnik

**Affiliations:** 1 Division of Neonatology, University of Washington, Seattle, WA 98195, USA; obrandon@uw.edu (O.C.B.); kcorry@uw.edu (K.A.C.); kdinucci@uw.edu (K.F.D.); moralejo@uw.edu (D.H.M.); sjuul@uw.edu (S.E.J.); tommyrw@uw.edu (T.R.W.); 2 Department of Chemical Engineering, University of Washington, Seattle, WA 98195, USA; zj39@uw.edu (Z.R.J.); schimek@uw.edu (N.S.); eanance@uw.edu (E.A.N.); 3 Department of Bioengineering, University of Washington, Seattle, WA 98195, USA; mmagoon@uw.edu (M.J.M.); pmjboyle@uw.edu (P.M.B.); 4 Institute on Human Development and Disability, University of Washington, Seattle, WA 98195, USA

**Keywords:** caffeine, neonatal, hypoxia–ischemia, organotypic slice culture, ferret

## Abstract

Brain injury after hypoxia–ischemia (HI) is the leading cause of morbidity and mortality in term and near-term neonates worldwide. The ferret is a promising translational model to study HI due to its gyrified brain and white-to-gray matter ratio that more closely resembles humans compared to rodents. Caffeine, an adenosine A2A receptor (A2AR) antagonist, shows neuroprotective potential after HI, but its effects have not been fully characterized. We sought to evaluate caffeine’s effect on neuronal cell death, cytotoxicity, and inflammatory and oxidative stress markers in a term-equivalent ferret organotypic brain slice model of HI. Slices were cultured for 72 h, exposed to two hours of oxygen–glucose deprivation (OGD), and randomized to OGD alone, OGD with caffeine (20 or 50 mg/L), or OGD with caffeine and an A2AR agonist. Healthy slices served as controls. Outcomes included global cell death, regional cell death, microglial morphology, and expression of inflammatory and oxidative stress genes (46–48 slices/group for cell death assays and 18 slices/group for imaging, balanced by sex). Caffeine 50 mg/L significantly reduced global cell death compared to OGD (*p* = 0.02), and this effect persisted despite co-administration of an A2AR agonist (*p* = 0.01), suggesting that protection was not primarily mediated through A2AR signaling. Caffeine also did not change regional pyknotic nuclei counts (*p* > 0.05). Caffeine altered microglial morphology, increasing the proportion of microglia with features characteristic of control conditions. OGD significantly increased expression of inflammatory and oxidative stress-related genes (*p* < 0.05) compared with control slices, whereas caffeine did not significantly alter gene expression. In summary, caffeine partially reversed global cell death after OGD and altered microglial morphology. Larger, higher-powered studies are needed to further investigate caffeine’s effects on neonatal HI.

## 1. Introduction

Brain injury after hypoxia–ischemia (HI) is the leading cause of neonatal morbidity and mortality worldwide [1]. Therapeutic hypothermia, the only currently approved treatment for HI, provides incomplete neuroprotection [2,3,4,5,6]. Caffeine is a promising neuroprotectant for HI as well as being inexpensive and shelf-stable, meaning it could also serve as an accessible neuroprotectant in low- and middle-income settings where therapeutic hypothermia has not shown benefit [7]. Neuroprotective effects of caffeine have been reported in preterm infants treated for apnea of prematurity and in rodent and ovine models of HI [8,9,10,11].

The mechanisms of caffeine neuroprotection, including antioxidant, anti-inflammatory, and anti-apoptotic properties have not been fully explored in the context of HI-induced injury [12,13]. As an adenosine A2A receptor (A2AR) antagonist, caffeine may mitigate A2AR-driven neuroinflammation via microglia cells after brain injury [14]. Microglia, the principal innate immune cells of the central nervous system, are key mediators of neuroinflammation following HI. Importantly, A2ARs are highly expressed on microglia and play a critical role in regulating their activation and cytokine production [15,16]. This positions microglia as a central cellular target through which caffeine may exert its neuroprotective effects. For example, in a rat model of HI-induced white matter damage, caffeine-mediated neuroprotection was reversed when it was combined with the A2AR agonist CGS-21680 hydrochloride hydrate at 2 mg/L [17]. However, the downstream effects of A2AR antagonism on oxidative stress and inflammatory signaling pathways remain incompletely defined. In particular, transcriptional markers of oxidative stress (e.g., heme oxygenase-1) and anti-inflammatory signaling (e.g., IL-10) may provide insight into how caffeine modulates injury responses following HI.

Caffeine has been shown to reduce brain tissue infarct in term rodent models of unilateral HI [12,18]. In neonatal rodent and ovine models of HI, caffeine reduced brain tissue loss, attenuated neuroinflammation, and improved functional outcomes such as cognition and motor coordination [9,14,17,19,20,21]. However, translation of these in vivo studies to larger animal models such as piglets have had mixed results with some showing no improvement in brain injury and potential toxicity at higher doses [22,23]. Toxicity and therapeutic ranges for caffeine dosing have mostly been described in preterm neonates with international standard of therapeutic range 5–30 mg/L and toxicity seen at levels ≥50 mg/L [24]. In term infants undergoing therapeutic hypothermia, goal concentrations are not well defined; however, current work in a phase I trial suggests a target of 15–25 mg/L, with the toxic range defined as >46 mg/L [25].

The ferret is a promising model in which to study HI due to its gyrified brain and white-to-gray matter ratio that more closely resembles humans compared to rodents [26,27,28,29]. As such, it may serve as an intermediate model between rodent and large-animal systems (e.g., piglet and sheep), providing an opportunity to help reconcile inconsistencies in neuroprotective outcomes observed across species. We developed an ex vivo model of hypoxic–ischemic injury in ferret brain slices using oxygen–glucose deprivation (OGD) [21,22]. In this study, we used the term-equivalent OGD-exposed ferret brain slice to determine the neuroprotective potential of caffeine in neonatal HI. Our primary hypothesis was that caffeine would reduce cellular injury, attenuate inflammatory and oxidative stress responses, and preserve microglial morphology following OGD. Our secondary mechanistic hypothesis was that these neuroprotective effects would be mediated, at least in part, through A2AR signaling, which we tested by co-administering an A2AR agonist. We examined the effect of caffeine on inflammation measured by PCR, neuronal cell death, microglial morphology across key brain regions in the ex vivo ferret brain as well as whether signs of toxicity present at high concentrations. Our slice culture model provides a unique opportunity to examine regional effects of HI with global and regional cell death analyses, allowing us to capture the heterogeneous and complex nature of HI across the brain [30,31,32]. Understanding these regional differences is critical, as injury patterns influence both functional outcomes and the efficacy of potential therapies.

## 2. Materials and Methods

### 2.1. Animal Care

All procedures in this study were conducted in full compliance with the Guide for the Care and Use of Laboratory Animals (National Institutes of Health, Bethesda, MD). Reporting followed relevant aspects of the ARRIVE (Animal Research: Reporting of In Vivo Experiments) guidelines. Animal handling and experimental protocols were approved by the University of Washington Institutional Animal Care and Use Committee (IACUC; protocol #3328-06). The University of Washington has an approved Animal Welfare Assurance (#A3464-01) on file with the NIH Office of Laboratory Animal Welfare (OLAW), is registered with the United States Department of Agriculture (certificate #91-R-0001), and is accredited by AAALAC International.

Ferret jills with cross-fostered kits were obtained from Marshall BioResources (North Rose, NY, USA) and delivered to the facility by postnatal day (P) 15 or earlier. Animals were housed in a centralized vivarium with free access to food and water. Housing conditions were standardized, including a 16 h light/8 h dark cycle, ambient temperature maintained between 61 and 72 °F (16–22 °C), humidity ranging from 30 to 70%, and 10–15 fresh air exchanges per hour. Every effort was taken to reduce animal distress.

### 2.2. Slice Preparation

We used an established ex vivo model of P21 whole-hemisphere brain slices that retain the cerebral architecture of in vivo models [33,34]. The experimental design is depicted in Supplemental Figure S1 [35]. Briefly, at P21, equivalent to term human gestation, ferret kits were first deeply anesthetized with 5% isoflurane. Animals were rapidly decapitated with a guillotine, and the brains were immediately extracted and transferred into ice-cold dissecting solution containing 0.64% *w*/*v* glucose (MilliporeSigma, Burlington, MA, USA), 100% Hank’s Balanced Salt Solution (HBSS, Thermo Fisher Scientific, Waltham, MA, USA), and 1% penicillin-streptomycin (Thermo Fisher Scientific, Waltham, MA, USA). Then, 300 µm whole-hemisphere slices were obtained using a Leica Vibratome (Leica Biosystems, Nussloch, Germany). Slices were plated into 35 mm, 0.4 µm pore membrane inserts (hydrophilic PET, CellTreat Scientific Products, Pepperell, MA, USA) in a six-well plate (USA Scientific Inc., Ocala, FL, USA) and cultured in 1 mL of 5% heat-inactivated horse-serum slice culture media (SCM), which contains 50% Minimum Essential Media (Thermo Fisher Scientific, Waltham, MA, USA), 45% HBSS, 1% GlutaMAX (Thermo Fisher Scientific, Waltham, MA, USA), and 1% penicillin-streptomycin. SCM was changed daily to ensure nutrients were replenished.

### 2.3. Oxygen–Glucose Deprivation

After 72 h in culture in a sterile CO_2_ incubator at constant temperature (37 °C), humidity, and CO_2_ (5%, balance air), slices were exposed to OGD for two hours to mimic HI. During the OGD period, SCM was replaced with glucose-free OGD medium (150 mM NaCl, 2.8 mM KCl, 1 mM CaCl_2_, and 10 mM HEPES in DI H_2_O, Thermo Fisher Scientific, Waltham, MA, USA). The medium was pre-warmed to 37 °C and equilibrated with N_2_ for 10 min at a flow rate of 5 L/min. Slices were then transferred into a chamber in the 37 °C incubator, which was flushed with N_2_ for 10 min at 5 L/min, and then subsequently sealed for 2 h. An oxygen monitor was used to ensure that oxygen did not rise above 1%. Following OGD, slices were removed from the chamber and the OGD media was replaced with either 5% SCM or SCM supplemented with caffeine (20 mg/L or 50 mg/L) or A2AR agonist (CGS-21680 hydrochloride hydrate, MilliporeSigma, Burlington, MA, USA) with caffeine 50 mg/L. For all experiments, the end of the OGD incubation was designated as time t = 0 h. Slices were then maintained in culture for an additional 24 h. Control slices were not exposed to OGD and underwent identical media changes (removal and replacement at the same time intervals) to match the handling of the 2 h OGD condition. Treatment groups are summarized in Supplemental Table S1. Supernatants were collected at 24 h.

### 2.4. Caffeine Dosage

Standard caffeine (Sagent Pharmaceuticals, Schaumburg, IL, USA) concentration was defined as 20 mg/L based on international standard of therapeutic range 5–30 mg/L [24]. A high concentration of 50 mg/L was based on described risk for toxicity ≥50 mg/L, although some studies note a wide therapeutic index, with safety shown at higher serum levels of 50–84 mg/L [36]. After OGD, slices were exposed to 1 mL of caffeine at the randomized concentration for 24 h.

### 2.5. Lactate Dehydrogenase Assay

Supernatant collected at the end of culture (t = 24 h) was immediately frozen at −80 °C and later thawed at room temperature for the lactate dehydrogenase (LDH) assay (Cayman Chemical, Ann Arbor, MI, USA). LDH, an enzyme released during cell membrane damage in response to cytotoxicity, is detected through coupled enzymatic reactions that generate formazan, which absorbs light within the 490–520 nm range. For the assay, 100 µL of thawed supernatant was plated in triplicate into a 96-well plate, followed by the addition of 100 µL chilled LDH reaction buffer per well. Plates were incubated at 37 °C for 30 min, after which absorbance was read at 490 nm (A490) on a UV-Vis spectrophotometer (Thermo Fisher Scientific, Waltham, MA, USA). Cumulative LDH release was determined by summing A490 values across the 24 h samples. Each group included *n* = 46–48 slices, evenly split by sex.

### 2.6. Immunofluorescent Staining

Slices were fixed with 10% buffered formalin and stored in 1 × phosphate-buffered saline (PBS) at 4 °C until imaging. Slices were stained for microglia using rabbit anti-ionized calcium-binding adaptor molecule 1 (rabbit anti-Iba1, Fujifilm, Santa Ana, CA, USA) and secondary antibody (AF-488 IgG goat anti rabbit, Invitrogen, Carlsbad, CA, USA) diluted in 1 × PBS containing 0.5% (*v*/*v*) Triton X-100, 1% (*v*/*v*) goat serum, and 1% (*w*/*v*) bovine serum albumin. Before imaging, slices were incubated in 1 mL of 5 μg/mL 4′,6-diamidino-2-phenylindole (DAPI; Invitrogen, Carlsbad, CA, USA) prepared in 1 × PBS for 15 min, followed by two washes in 1 × PBS. Using a Nikon A1R confocal microscope with 40× objective, a minimum of *n* = 18 slices per condition were imaged with an equal sex split. Each slice had 3–5 imaging locations captured per region of interest including: cortex, subcortical white matter, corpus callosum, thalamus, basal ganglia, and hippocampus.

### 2.7. Automated Nuclei Counting

We developed a custom code (Python v3.10.11) for automatically identifying nuclei and classifying them as pyknotic or non-pyknotic in this injury model, optimized from a previously published methodology for automatically counting nuclei in organotypic ferret brain slices [35]. Briefly, two-dimensional DAPI-stained histology images with 512 × 512 pixels, 0.863 × 0.863 μm^2^ per pixel were obtained (Supplemental Figure S2A). The DAPI channel was preprocessed to remove bright spots with a radius ≤1 pixel, the baseline intensity was corrected with the rolling ball algorithm (scikit-image) [37,38], and pixels with a relative intensity <10% were masked (Supplemental Figure S2B,C). Nuclei were detected with the Laplacian of Gaussian method (scikit-image) parameterized as follows: min_sigma = 2.2 µm ≈ 2.5 pixels, max_sigma = 4.2 µm ≈ 4.9 pixels, num_sigma = 21, threshold = 0.5% of the maximum pixel intensity, threshold_rel  =  0, and overlap = 0.7 [37]. Watershed (scikit-image) segmentation further delineated boundaries between clusters of nuclei (Supplemental Figure S2D) [37].

Seven previously described nuclear parameters were calculated only in the DAPI channel: area, Crofton four-direction perimeter approximation (scikit-image) [37], ideal radius, eccentricity, average normalized intensity, total normalized intensity, and weighted intensity (by distance from the center of the nucleus). The sigma parameter describing each nucleus’s Gaussian curve of best fit when it was originally identified by the Laplacian of Gaussian method was also noted. Nuclei were filtered to ensure ideal radius >2.25 µm, average normalized intensity >5%, and at least 16 pixels per nucleus. Consistent with manual nuclei counts, nuclei touching an edge of the image were removed. These parameters and criteria were selected to yield apparently reliable image segmentations, reducing false positives from large nucleosomes or debris, before nuclei counts were used for subsequent analysis. A random forest classifier (scikit-learn [39]) was trained on manually annotated images from a different dataset to identify pyknotic nuclei using only the eight measurements derived from the DAPI channel, as previously described [35]. The random forest classifier was applied to identify pyknotic nuclei in this entirely new dataset without further modification (Supplemental Figure S2E). The researcher who developed the code/random forest classifier and generated all counts was fully blinded to treatment groups and outcomes.

### 2.8. Microglial Morphological Analysis

We used the previously published machine learning Visual Aided Morpho-Phenotyping Image Recognition (VAMPIRE) pipeline to determine five shape modes (SMs) that described the distribution of microglia morphologies [34]. To process the data for VAMPIRE, the Iba1 channel was extracted from each image, and a Li threshold (scikit-image) was used to create a binary mask of the cells in each image. The region props function from scikit-image was used to calculate the geometric properties of each cell using the binary masks. Images were divided into train and test splits at an 80:20 ratio to train the VAMPIRE model. To prevent data leakage, images from the same slice were put into only the training or only testing dataset. In the VAMPIRE pipeline, 50 points were selected equidistantly around the perimeter of the mask of each cell to generate two-dimensional contours, which were then normalized and aligned. Principal component analysis was used for dimensionality reduction in the contours. The number of principal components to keep was chosen to capture 95% of the variance in the dataset. The eigen-shapes from the kept principal component were used to create a reconstruction of each cell. The reconstructed shapes of the cells from the training set were used to train a K-means clustering algorithm to separate the shapes into 5 clusters, which were assigned shape mode 1–5. The trained model was then used to predict the shape mode for each cell in the testing dataset.

### 2.9. RNA Extraction and qRT-PCR

At 24 h, slices were carefully removed from membranes and stored at −80 °C. RNA extraction from tissue was performed with the Qiagen RNeasy Kit (Qiagen, Hilden, Germany) in accordance with the manufacturer’s guidelines. RNA quantity and purity were assessed using a Nanodrop (Thermo Fisher Scientific, Waltham, MA, USA), with samples included if the A260/A280 ratio was ≥1.8. RNA was reverse-transcribed into cDNA using the High-Capacity RNA-to-cDNA kit (Applied Biosystems, Waltham, MA, USA) and diluted to 20 ng/µL. Real-time quantitative reverse transcription-polymerase chain reaction (qRT-PCR) was conducted with PowerUp SYBR Green Master Mix (Applied Biosystems, A25741, Waltham, MA, USA) according to manufacturer instructions. RNA was extracted from the entire organotypic slice; therefore, qRT-PCR measurements represent whole-slice gene expression rather than region-specific transcriptional changes. Custom primers targeting ferret-specific genes were used to quantify expression of CASP, EDEM, GCLM, HMOX, IL10, and BIP. To evaluate pathways implicated in HI and previously associated with caffeine-mediated neuroprotection, we selected genes representing apoptosis (CASP), endoplasmic reticulum stress (BIP, EDEM), oxidative stress (HMOX, GCLM), and inflammation (IL10) [17,40,41]. Relative expression was calculated using the ΔCt method normalized to GAPDH, with ΔΔCt determined against control slices. Data represent *n* = 18 slices per condition, balanced by sex.

### 2.10. Statistical Analysis

Slices were collected from two independent experiments. To account for potential inter-experiment variability, all graphs across experiments analyzed were relative to the control median within each experiment. Regional analyses were normalized to the control median of the corresponding region. Kruskal–Wallis tests with Dunn’s post hoc correction for multiple comparisons were used to evaluate differences across treatments in LDH and qRT-PCR. To assess differences in cell death by pyknotic nuclei or assess the number of microglia, linear mixed-effect models were used with fixed effect for region (in global analyses) and random effect by slice and experiment.

A Mantel-Haezsel (multivariable Chi-square) test was used to assess the distribution of shape modes between treatment groups, adjusting for the effect of brain region. To assess differences in microglial morphology by shape modes, logistic mixed-effect models with a fixed effect for region and random effect by slice and experiment were used to assess each shape model individually. When analyses were restricted to a single region, the fixed effect for region was not included in the model.

Graphical network analysis identified associations among interrelated variables by estimating a precision matrix (inverse correlation matrix), which accounts for the influence of all other variables in the system. Partial correlations were obtained by inverting the correlation matrix of the variables, and relationships were retained if their 95% confidence intervals did not include zero [35,42].

A probability (*p*) value less than 0.05 was considered significant for all analyses. All statistical analyses were performed in R Version 4.2.1 (Vienna, Austria) [43]. Additional figures were made in GraphPad Prism version 10.5.0 (GraphPad software, San Diego, CA, USA).

## 3. Results

### 3.1. High-Dose Caffeine Reduces LDH, While OGD Increases Neuronal Cell Death

**Figure 1:**
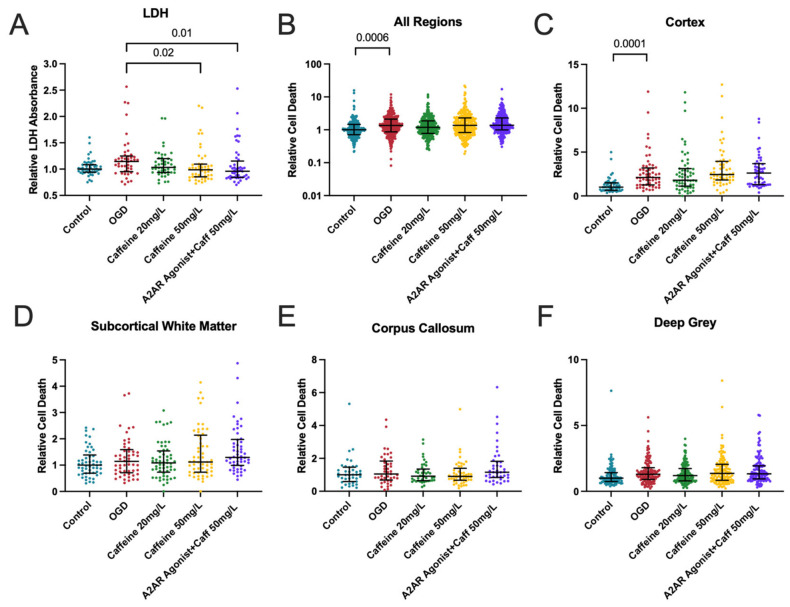
Cell death across treatments. Lactate dehydrogenase (LDH; (**A**)), neuronal cell death globally (**B**), in the cortex (**C**), subcortical white matter (**D**), corpus callosum (**E**), and deep gray matter (**F**) shown relative to the control median in each region per experiment. Caffeine 50 mg/L both with (*p* = 0.01) and without (*p* = 0.02) the agonist resulted in significantly reduced LDH release. Globally and in the cortex, slices exposed to oxygen–glucose deprivation (OGD) had significantly higher relative cell death compared to controls (*p* = 0.0006, *p* = 0.0001, respectively). Caffeine at 20 mg/L, 50 mg/L, or when combined with the A2AR agonist had similar relative cell death compared to OGD. Deep gray matter included basal ganglia, thalamus, and hippocampus. Kruskal–Wallis test with Dunn’s post hoc correction for multiple comparisons used for LDH. Linear mixed-effect model used with fixed effect for region (in global and deep gray analyses) and random effect by slice and experiment. Some subregions exclude 1–4 data points on graph for visualization. *p* < 0.05 considered significant.

Caffeine 50 mg/L (*p* = 0.02) and caffeine 50 mg/L + A2AR agonist (*p* = 0.01) resulted in significantly lower LDH release compared to OGD (Figure 1A). Using pyknotic nuclei counts, OGD significantly increased cell death relative to control globally (*p* = 0.0006; Figure 1B) and in the cortex (*p* = 0.0001; Figure 1C) OGD did not increase cell death in the subcortical white matter (Figure 1D), corpus callosum (Figure 1E), or in deep gray matter regions (Figure 1F). There were no differences in cell death across treatments in deep gray matter regions including the basal ganglia, thalamus, and hippocampus (Supplemental Figure S3). Caffeine at 20 mg/L, 50 mg/L alone, or combined with the A2AR agonist did not significantly alter cell death from OGD globally or regionally (Figure 1).

### 3.2. Caffeine Increases Number of Microglia and Alters Morphology

**Figure 2:**
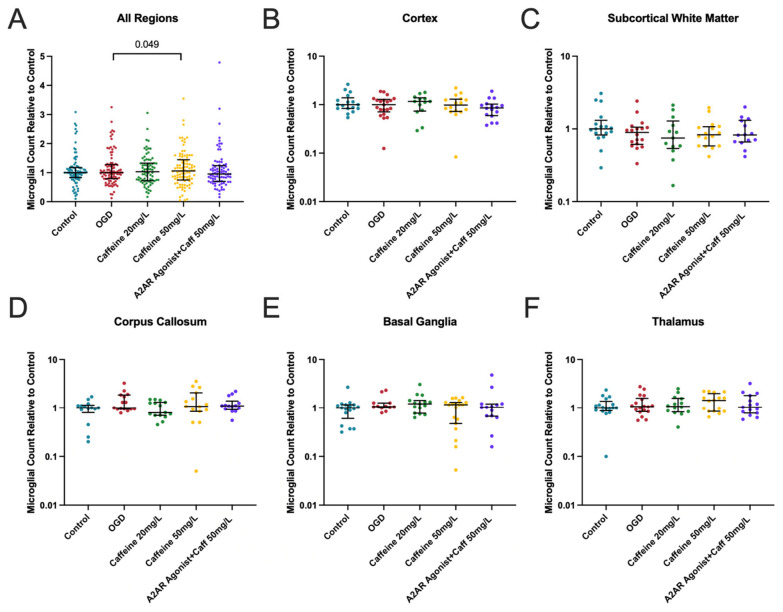
Microglial counts by treatment in all regions (**A**), cortex (**B**), subcortical white matter (**C**), corpus collosum (**D**), basal ganglia (**E**), and thalamus (**F**). Microglia count shown relative to the control median in each region per experiment. Across all regions combined, caffeine at 50 mg/L resulted in significantly more microglia compared to oxygen–glucose deprivation (*p* = 0.049). No differences in number of microglia were seen in any subregion. Linear mixed-effect model used with fixed effect for region and random effect by slice and experiment.

Caffeine 50 mg/L had significantly higher microglia per image compared to control (*p* = 0.049, Figure 2A). No differences in number of microglia were seen across regions (Figure 2B–F).

**Figure 3:**
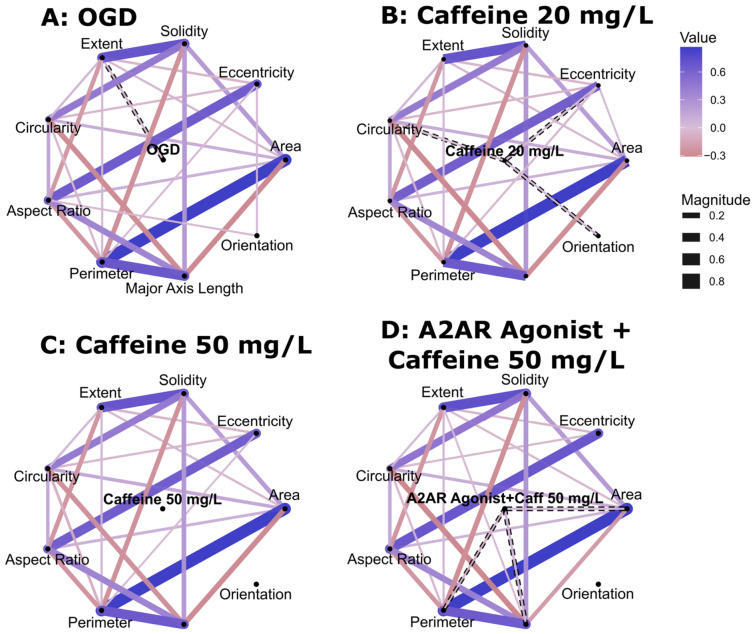
Graphical network analyses of microglial morphological parameters across oxygen–glucose deprivation (OGD; (**A**)), caffeine 20 mg/L (**B**), caffeine 50 mg/L (**C**), and A2AR agonist + caffeine 50 mg/L (**D**). OGD was significantly negatively associated with extent. Caffeine at 20 mg/L was significantly negatively associated with circularity and eccentricity and positively associated with orientation. Caffeine at 50 mg/L was not associated with microglial parameters. A2AR agonist with caffeine at 50 mg/L was significantly negatively associated with perimeter and positively associated with major axis length and area. All associations are relative to OGD. Lines indicate significant associations between parameters from negative (red) to positive (blue). Thickness of the line indicates the magnitude of the association. Significant associations with center node outlined with dashes for clarity.

Looking at microglial morphology across groups, OGD was associated with significantly decreased extent (Figure 3A), consistent with a shift toward a less ramified morphology typical of activated microglia. Caffeine at 20 mg/L was significantly associated with decreased circularity and eccentricity and positively associated with orientation (Figure 3B), suggesting a more elongated and polarized morphology that may reflect partial preservation of microglial process structure. In contrast, caffeine at 50 mg/L was not associated with distinct microglial parameters compared to OGD (Figure 3C), indicating little morphological recovery relative to injury alone. Treatment with an A2AR agonist in combination with caffeine at 50 mg/L was significantly associated with increased area and major axis length and decreased perimeter (Figure 3D), morphological features consistent with a more enlarged and less ramified cell body often observed during microglial activation.

To characterize how HI injury and caffeine treatment influenced microglial morphology, we performed quantitative morphometric analysis of Iba1^+^ microglia across treatment groups and brain regions. Microglia were classified into five discrete shape modes, each representing a distinct combination of morphometric features. SM1 was associated with increased aspect ratio, indicating more elongated microglial morphology (Supplemental Figure S4A). In contrast, SM2 was associated with decreased solidity and aspect ratio with increased eccentricity and circularity, suggesting a shift toward a more rounded cell shape (Supplemental Figure S4B). SM4 and SM5 were associated with decreased aspect ratio and eccentricity, respectively, consistent with progressively less elongated and more circular microglial morphology (Supplemental Figure S4C,D).

**Figure 4:**
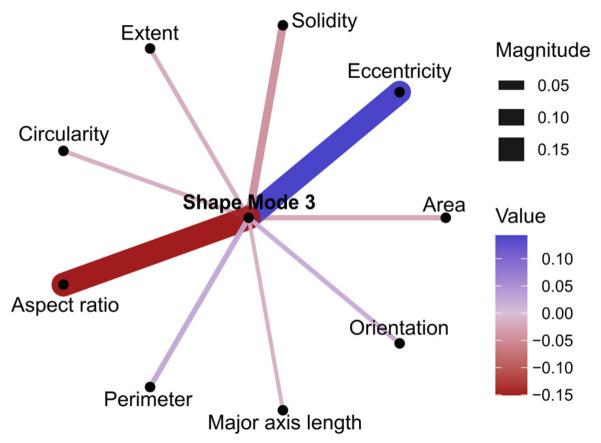
Shape mode 3 and association with microglial parameters. Lines indicate significant associations between parameters from negative (red) to positive (blue). Thickness of the line indicates the magnitude of the association. Only lines connected to SM3 shown. SM3 was most significantly associated with decreased aspect ratio and increased eccentricity.

Overall, the distribution of microglia SMs significantly differed between treatment groups after adjusting for region (*p* = 0.006). Looking at specific treatments after adjusting for region, control and caffeine at 20 mg/L had a significantly larger portion of SM3 compared to OGD (Supplemental Figure S5). SM3 was significantly associated with decreased aspect ratio (a shorter cell body) and increased eccentricity (more elliptical shape) compared to the other SMs (Figure 4). This morphology reflects a shift toward a less ramified microglial state, which is commonly associated with an activated response to injury.

Regionally, no differences in SM proportions were observed in the thalamus (Supplemental Figure S6A). In the basal ganglia, SM1 was reduced in the caffeine 50 mg/L group, while both 20 mg/L and 50 mg/L caffeine treatments were associated with significantly lower SM2 and higher SM5 proportions compared to OGD (Supplemental Figure S6B). In the hippocampus, caffeine at 50 mg/L, with or without the A2AR agonist, resulted in fewer SM1 compared to OGD, indicating a shift toward more circular microglial morphology (Supplemental Figure S6C). In addition, the combination of caffeine 50 mg/L and the A2AR agonist produced lower SM3 and higher SM5 proportions than OGD (Supplemental Figure S6C). In the subcortical white matter, SM2 and SM3 were significantly higher, while SM5 was lower, in the caffeine 20 mg/L group compared to OGD (Supplemental Figure S6D). In the corpus callosum, SM1 was significantly higher in both caffeine treatment groups, whereas SM5 was significantly lower in the caffeine 20 mg/L group compared to OGD (Supplemental Figure S6E). Overall, in white matter regions, caffeine increased elongated morphologies (SM1-SM3) and reduced SM5, suggesting preservation of less rounded microglial states. In the cortex, only SM3 was significantly higher in the caffeine 50 mg/L compared to OGD (Supplemental Figure S6F).

### 3.3. OGD Increases Genetic Markers of Oxidative Stress and Inflammation

**Figure 5:**
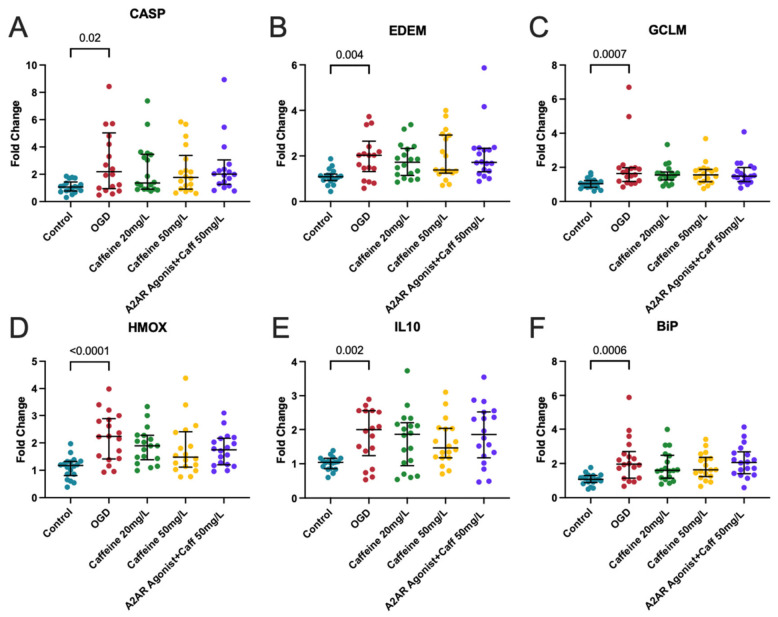
qRT-PCR markers of oxidative stress and inflammation, including CASP (**A**), EDEM (**B**), GCLM (**C**), HMOX (**D**), IL10 (**E**), and BiP (**F**). Oxygen–glucose deprivation (OGD) significantly increased all measured gene expressions compared to control slices. While not significant, there is a dose-dependent pattern of caffeine at 50 mg/L altering gene expression more than caffeine at 20 mg/L, and caffeine at 20 mg/L altering gene expression more than OGD (no caffeine exposure) for HMOX and IL10 expression. Some genes exclude 1–2 data points on graph for visualization. Kruskal–Wallis test used, and *p* < 0.05 considered significant.

To investigate the molecular mechanisms underlying these cellular changes, we next assessed transcriptional markers of oxidative stress and inflammation using qRT-PCR. All six genes tested (CASP3, EDEM, GCLM, HMOX, IL10, and BiP) were significantly upregulated in OGD compared to control conditions, and their expression was not altered by caffeine treatment (Figure 5). Although not statistically significant, HMOX and IL10 expression demonstrated a dose-dependent reduction, with caffeine at 50 mg/L lowering expression more than caffeine at 20 mg/L, and 20 mg/L lowering expression below that of OGD without caffeine exposure (Figure 5D,E).

## 4. Discussion

Perinatal hypoxic–ischemic injury remains a major cause of neonatal morbidity and mortality, and new therapeutic strategies are urgently needed to complement or enhance existing treatments. The neuromodulator caffeine, a widely used neonatal therapy for apnea of prematurity, has been proposed to provide neuroprotective effects through adenosine receptor modulation and antioxidant pathways by inhibiting ferroptosis, reducing oxidative stress, and decreasing inflammatory cytokine production [44]. We tested the effects of caffeine in an ex vivo ferret model of HI to evaluate its impact on cell death, oxidative stress, microglial morphology, and gene expression, as well as to determine if toxicity occurred at the cellular level with higher doses. Although there was no evidence of overt toxicity with either standard (20 mg/L) or high (50 mg/L)-dose caffeine, the potential to mitigate cellular injury differed between the two doses in this study. Interestingly, microglial morphology at 50 mg/L resembled that seen following OGD alone, suggesting that higher caffeine exposure may not fully attenuate injury-associated microglial activation. In contrast, the improvement observed at 20 mg/L may reflect a dose range that is protective without overstimulating microglia, which express relatively high densities of adenosine receptors. The improved LDH signal at 50 mg/L may therefore reflect protection of other cell populations, producing a more global effect despite potential microglial vulnerability.

Caffeine at 50 mg/L, with and without the A2AR agonist, decreased cellular death as measured by LDH. However, cell death measured by pyknotic nuclei was not ameliorated by caffeine at either 20 mg/L or 50 mg/L. Similarly, caffeine at either dose did not reverse expression of genes related to oxidative stress and inflammation that were elevated after OGD. One potential explanation for this discrepancy is that LDH and pyknosis capture distinct aspects of injury. LDH release reflects global loss of membrane integrity and cytotoxicity arising from multiple cell populations, including neurons, astrocytes, oligodendrocytes, and microglia, whereas pyknotic nuclei represent irreversible nuclear condensation associated with dying cells. Thus, caffeine may preserve membrane integrity or reduce secondary cytotoxic injury without preventing established neuronal cell death. This interpretation is also consistent with our finding that high-dose caffeine reduced LDH release despite producing microglial morphology similar to OGD, suggesting that protection may have occurred predominantly in non-neuronal cell populations. This finding is not consistent with prior studies in rodent models, where caffeine has been found to have antioxidant, anti-inflammatory and anti-apoptotic neuroprotective properties [14,17,19,41]. This lack of cellular response in our study may be due to timing of caffeine administration as it relates to injury. For example, caffeine given the day prior to injury and up to 3 days after injury demonstrated inhibition of transcription of the proinflammatory TNF-alpha and IL-1beta as well as promoted the transcription of anti-inflammatory factors 1L-10 and TGF-Beta [17].

Caffeine works as an antagonist for A1 adenosine receptors (A1AR) and A2AR; however, it operates as a non-specific AR antagonist and binds to varying degrees to all three of the ARs (A1, A2, A2b) [45]. A1R and A2AR are expressed in pre- and postsynaptic sites of neurons and are present in astrocytes, oligodendrocytes, and microglia [12,46]. A2AR has been implicated in neuroinflammation such that its expression in microglia cells is increased after brain injury [14]. These receptors are found in high concentrations in the brain, particularly the hippocampus and neocortex [45]. In our study, co-administration with the A2AR agonist did not meaningfully alter caffeine’s effects, which may reflect that caffeine’s neuroprotection is not driven primarily by A2AR antagonism. Instead, caffeine’s neuroprotective effects may reflect engagement of A1 receptor-dependent mechanisms and other intracellular pathways. Supporting this, a preclinical rat pup model of unilateral HI demonstrated that caffeine’s actions are mediated by AMP-activated protein kinase (AMPK) and mammalian target of rapamycin (mTOR) signaling [19]. Caffeine has also been shown to potentiate neural plasticity at the level of N-Methyl-D-Aspartate (NMDA) receptors as well as enhance activity-dependent brain-derived neurotrophic factor (BDNF) expression in cortical neurons, which may contribute to the neurological benefits [45].

When looking more specifically at microglia, the principal innate immune effector cells of the central nervous system, whose morphology and function rapidly adapt to fluctuations in the brain [47], we found caffeine at 50 mg/L significantly increased the number of microglia compared to OGD. Although increased microglial abundance may indicate an inflammatory response to hypoxic–ischemic injury, caffeine at 50 mg/L did not show distinct microglial morphological changes compared with OGD alone. In contrast, caffeine at 20 mg/L altered microglial morphology, resulting in a similar SM distribution to control slices after OGD. Specifically, OGD had decreased extent, suggesting a shift away from surveillant morphology toward injury-reactive microglia. Caffeine at 20 mg/L had decreased circularity and eccentricity and was positively associated with orientation. This is consistent with modulated or restrained activation, rather than amoeboid transformation. In previous research, microglia became more ameboid following OGD with decreased area and increased circularity [34]. We also found that SM3 occurred more frequently in control and 20 mg/L caffeine-treated slices compared to OGD, suggesting that OGD reduced the proportion of this morphological profile whereas caffeine partially protected against this loss. SM3 was linked to morphometric signatures of a less ramified phenotype (lower aspect ratio, higher eccentricity), consistent with a shift away from highly branched surveillance states. This altered microglial activity is also consistent with in vivo studies. For example, caffeine treatment at 20 mg/kg/day in term rat pups subjected to unilateral common carotid artery ligation and hypoxia was associated with suppression of NLRP3 inflammasome activation, reduced Iba1^+^ microglial activation, and a shift away from proinflammatory signaling, accompanied by decreased expression of CD8 and iNOS [17]. In a term mouse HI model, in both the cortex and striatum, caffeine reduced the density of Iba1^+^ amoeboid microglia and led to a smaller soma size compared to vehicle-treated mice similar to our results [14].

Within the brain, caffeine acts as a central nervous system stimulant by blocking adenosine receptors, which secondarily modulates the activity of other neurotransmitter systems, including gamma-aminobutyric acid (GABA), dopamine, serotonin, noradrenaline, and acetylcholine [36]. Caffeine can also act through inhibition of phosphodiesterase, preventing the breakdown of cyclic adenosine monophosphate (cAMP) and stimulating the central nervous system, as well as through inhibition of voltage-sensitive calcium channels, which can reduce neurotransmission [36]. These adenosine receptors are also expressed outside the brain, leading to important peripheral effects of caffeine; notably, A2A receptors are present at moderate concentrations in the lung. Some key cardiopulmonary functions of caffeine include stimulation of myocardium, increasing heart rate, cardiac output, and arterial blood pressure as well as increasing respiratory rate, diaphragmatic function, and pulmonary blood flow, which may play a part in blunting hypoxic events that the brain experiences [48,49]. Therefore, it is important to emphasize our study only captures the cellular effects of caffeine on the brain after HI, which is an incomplete model of caffeine effects in vivo.

From a translational perspective, our findings support continued investigation of caffeine as an adjunctive therapy for neonatal HI while emphasizing the need for ongoing research into the optimum dose, timing, and toxicity profile. Although the standard dosing of caffeine used in our experimental design of 20 mg/L (therapeutic range 5–30 mg/L in neonates) aligns with current neonatal use in traditionally more preterm infants with apnea of prematurity [36,50], this dosing did not translate to a measurable neuroprotective effect in our study design. Whereas the higher dosing of 50 mg/L (therapeutic range 50–84 mg/L in neonates) reduced global cytotoxicity, the lack of effect on regional neuronal cell death and transcriptional markers suggests that caffeine alone may provide only partial neuroprotection and must be balanced with ill-defined toxicity profiles at this dosing in neonates. Future in vivo studies are needed to determine whether these cellular effects translate into improved neurological outcomes, particularly in combination with therapeutic hypothermia as well as characterizing caffeine’s toxicity profile on neuronal cells.

This study had several strengths, including the assessment of multiple pathways of cell death, oxidative stress, and microglial morphology. Use of the ferret, a gyrencephalic mammal, provides greater translational relevance compared to rodent models. The slice culture system also partially preserves the three-dimensional brain architecture, enabling regional analyses that are particularly valuable given the heterogeneous nature of HI. Key limitations of this study include the inability of slice culture to capture peripheral contributions, such as cardiopulmonary effects, that may underlie the neuroprotective properties of caffeine reported in vivo. In addition, caffeine was tested at a single timepoint immediately after OGD, with outcomes measured only at 24 h. This design may not have captured the temporal dynamics of caffeine’s neuroprotective effects, as anti-inflammatory, antioxidant, and cell survival pathways may emerge earlier or later after injury. Caffeine’s neuroprotection may also be largely dependent on the timing of administration as previous research has shown neuroprotection with pretreatment or treatment at the time of injury [20,51,52]. OGD resulted in mild injury in this study and more severe models including longer OGD periods or intermittent hypoxia or prolonged nutrient deprivation may be needed. In addition, this study did not include caffeine-treated control slices without OGD. Such groups would help distinguish baseline effects of caffeine from its effects following HI and should be included in future studies. Finally, while experiments were sex-balanced, the study was not sufficiently powered to detect sex-dependent treatment effects.

## 5. Conclusions

Using our ex vivo ferret HI model, treatment with 50 mg/L caffeine, either alone or in combination with an A2AR agonist, reduced global cell death as indicated by LDH release. OGD increased nuclear pyknosis; these effects were not improved by caffeine. At the lower 20 mg/L dose, caffeine produced a microglial shape-mode profile resembling control tissue. Overall, we found that caffeine exhibited dose-dependent effects, with higher doses suppressing LDH release while lower doses more strongly influenced microglial measures, suggesting that different injury-related pathways may be differentially modulated across doses. In addition, OGD upregulated transcripts linked to oxidative stress and inflammatory signaling, and these gene expression changes were not significantly affected by caffeine exposure. Higher-powered studies across a range of preclinical models are warranted to further explore the effects of caffeine on HI.

## Data Availability

The data described in the manuscript and/or analyzed during the current study will be made available upon reasonable request from the corresponding author. The publicly available code for nuclei counts is available at https://github.com/MattM719/NeuroCellCounter. Accessed on 1 June 2026.

## Supplementary Materials

The following supporting information can be downloaded at https://www.mdpi.com/article/10.3390/neurosci7040079/s1, Figure S1: Overview of experimental design; Figure S2: Example nuclei counting code; Table S1: Summary of conditions and treatment groups; Figure S3: Neuronal cell death across treatment in deep gray matter regions; Figure S4: Shape modes and association with microglial parameters; Figure S5: Proportion of global microglial shape mode by treatment; Figure S6: Regional analyses of microglial shape modes.

## Abbreviations

The following abbreviations are used in this manuscript:

ADCE	Average daily caffeine exposure
aEEG	Amplitude-integrated EEG
AMPK	AMP-activated protein kinase
AR	Adenosine receptor
ARRIVE	Animal Research: Reporting of In Vivo Experiments
BDNF	Brain-derived neurotrophic factor
BUN	Blood urea nitrogen
cAMP	Cyclic adenosine monophosphate
DAPI	4′,6-diamidino-2-phenylindole
GABA	Gamma-aminobutyric acid
HBSS	Hank’s Balanced Salt Solution
HEPES	4-(2-hydroxyethyl)-1-piperazineethanesulfonic acid
HI	Hypoxia–ischemia
IACUC	Institutional Animal Care and Use Committee
LDH	Lactate dehydrogenase
MRI	Magnetic resonance imaging
mTOR	Mammalian target of rapamycin
NIH	National Institutes of Health
NMDA	N-Methyl-D-Aspartate
OGD	Oxygen–glucose deprivation
OLAW	Office of Laboratory Animal Welfare
P	Postnatal day
PBS	Phosphate-buffered saline
p	Probability
qRT-PCR	Quantitative reverse transcription-polymerase chain reaction
SCM	Slice culture media
SM	Shape mode
VAMPIRE	Visual Aided Morpho-Phenotyping Image Recognition
