# Supplementary material for: Effect of Caffeine on Cell Death, Oxidative Stress, and Microglial Morphology in a Ferret Organotypic Brain Slice Model of Hypoxia–Ischemia
Source: NeuroSci. 2026 Jul 10;7(4):79. doi: 10.3390/neurosci7040079 (PMC13398045; doi:10.3390/neurosci7040079)
Supplement: Supplementary file 1 [file neurosci-07-00079-s001.zip › neurosci-4379263-supplementary.pdf]

NeuroSci

MDPI

Supplemental Material

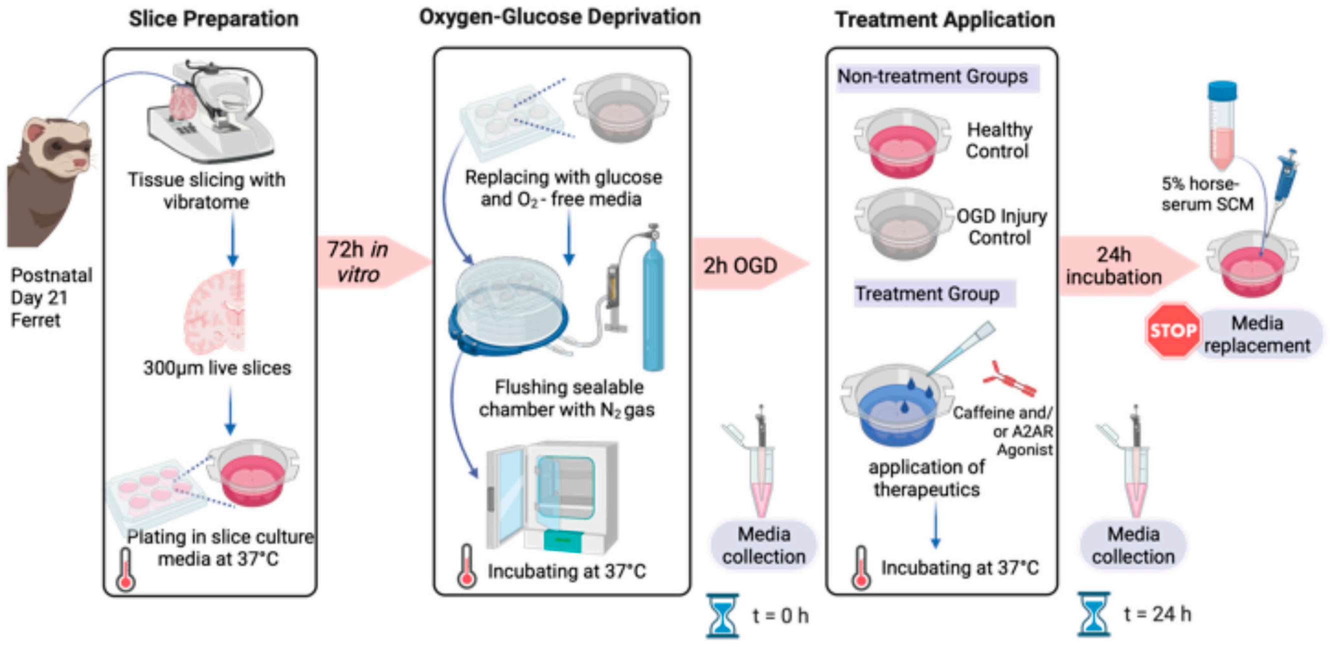

Supplemental Figure S1. Overview of experimental design. OGD: oxygen-glucose deprivation. SCM: slice culture media. Figure modified from Jin et al.

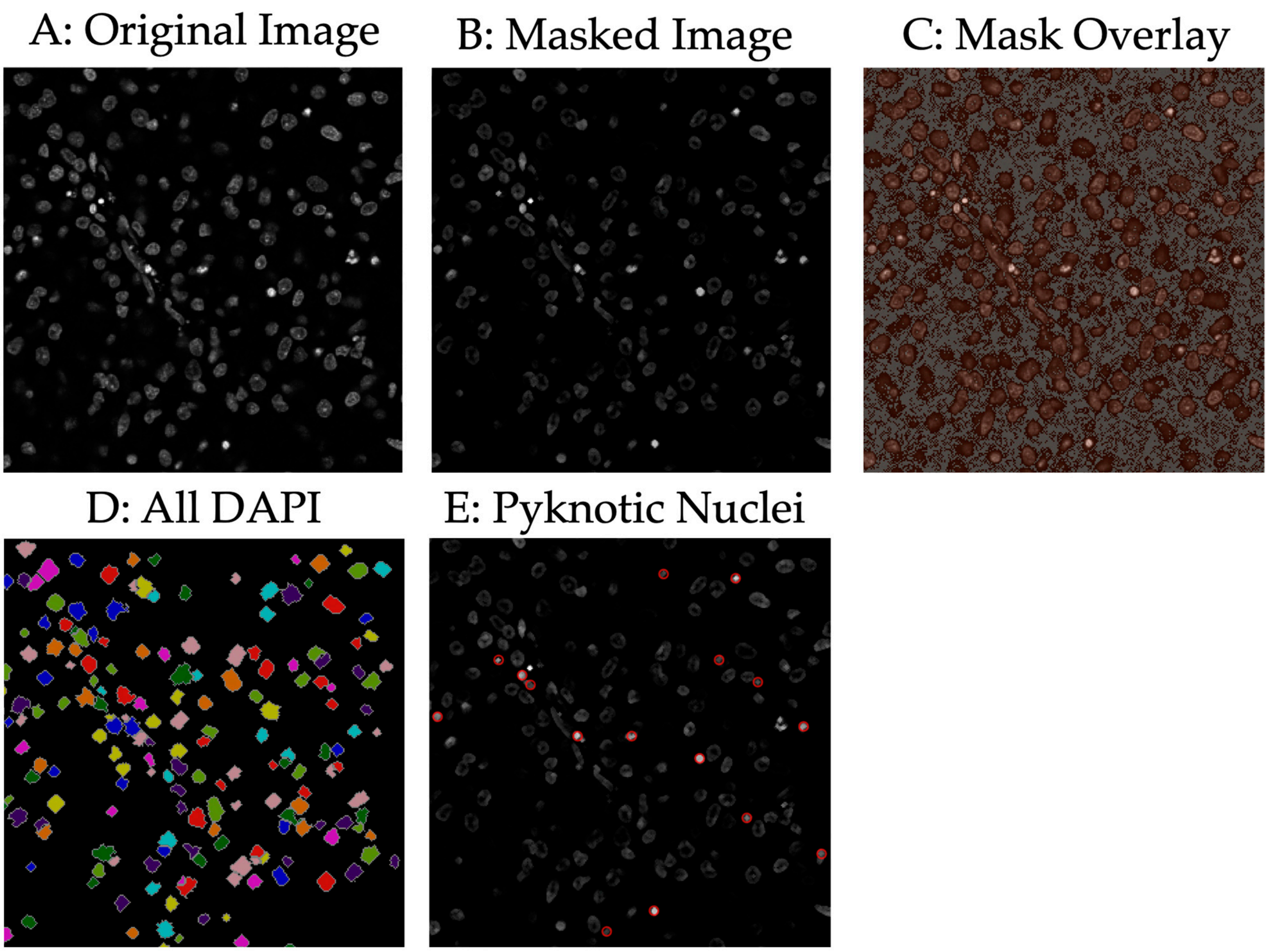

Supplemental Figure S2. Example nuclei counting code. Raw DAPI-stained images (**A**) were pre-processed and masked above 10% of the maximum intensity to reduce background noise (**B, C**). Nuclei were identified with the Laplacian of Gaussian method (**D**) before morphology and intensity-based measurements were used to classify each nucleus as pyknotic or non-pyknotic with a random forest classifier (**E**). Red circled cells indicate pyknotic nuclei (**F**).

Supplemental Table S1. Summary of conditions and treatment groups.

**Condition**	**OGD**<br>**After 72 hours rest, then 2 hours OGD**	**Treatment**<br>**0-hour after OGD treatment; at 24 hours collect for analyses**
Control	No	None
Oxygen-Glucose Deprivation (OGD)	Yes	None
Caffeine 20 mg/L	Yes	Caffeine 20 mg/L
Caffeine 50 mg/L	Yes	Caffeine 50 mg/L
CGS 2 mg/L + Caffeine 50 mg/L	Yes	CGS 2 mg/L + Caffeine 50 mg/L

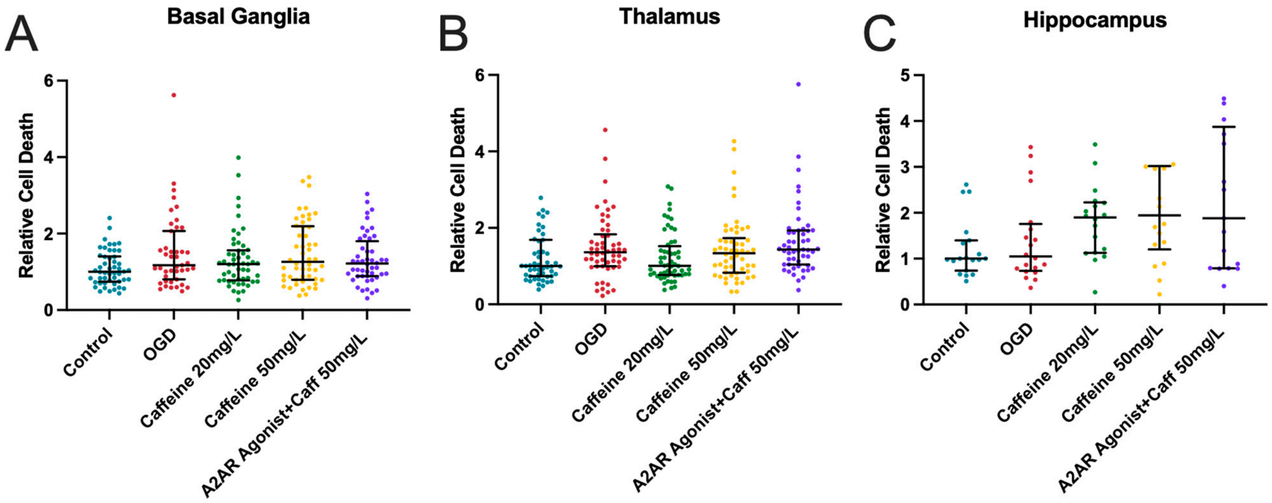

Suplemental Figure S3. Neuronal cell death across treatments in deep gray matter regions. Basal ganglia (**A**), thalamus (**B**), and hippocampus (**C**) shown relative to the control median in each region per experiment. There were no differences in neuronal cell death by treatment in these regions. Linear mixed effect model used with random effect by slice and experiment. Some subregions exclude 1-4 data points on graph for visualization. $p<0.05$ considered significant. OGD: oxygen-glucose deprivation.

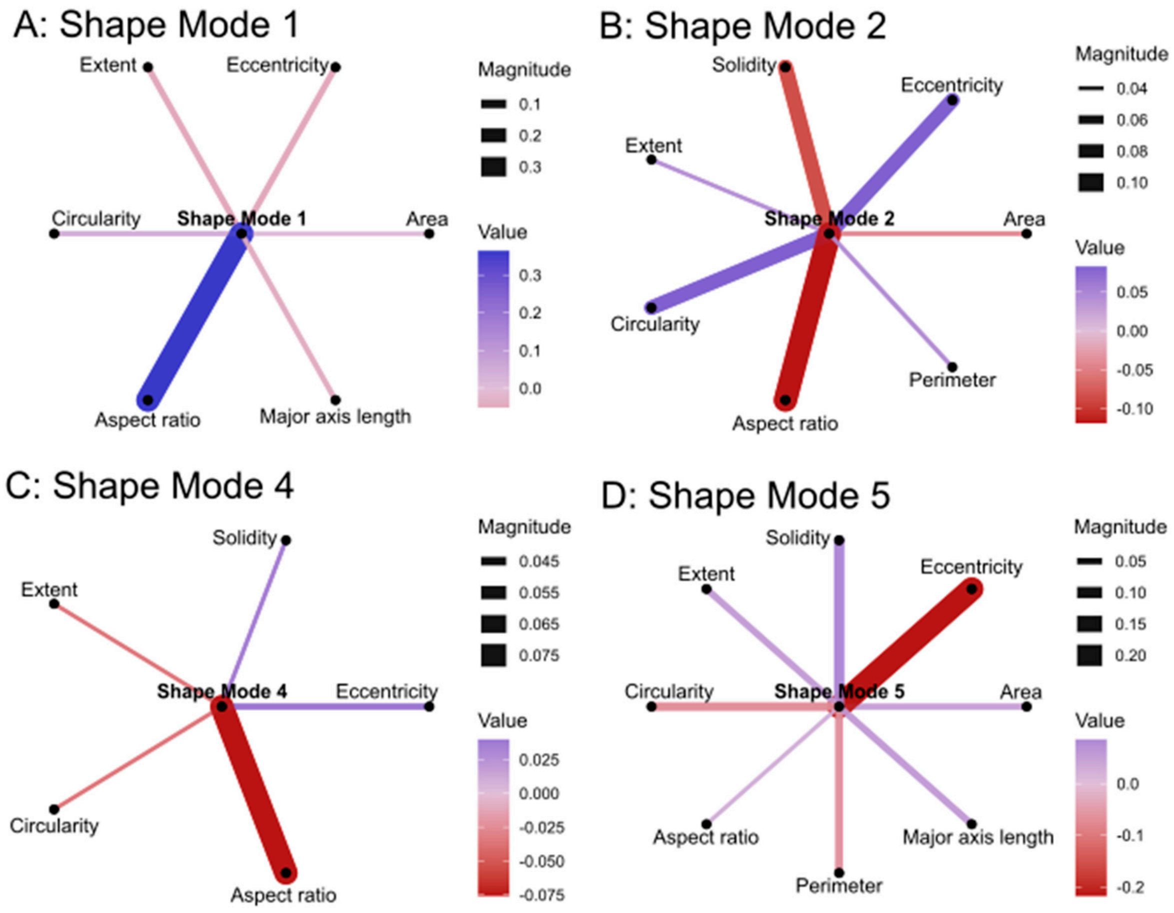

Supplemental Figure S4. Shape modes (SM) 1 (**A**), 2 (**B**), 4 (**C**), & 5 (**D**) and association with microglial parameters. Lines indicate significant associations between parameters from negative (red) to positive (blue). Thickness of the line indicates the magnitude of the association. SM1 was most significantly associated with increased aspect ratio. SM2 was most significantly associated with deceased solidity and aspect ratio and increased eccentricity and circularity. SM4 was

most significantly associated with decreased aspect ratio. SM5 was most significantly associated with decreased eccentricity.

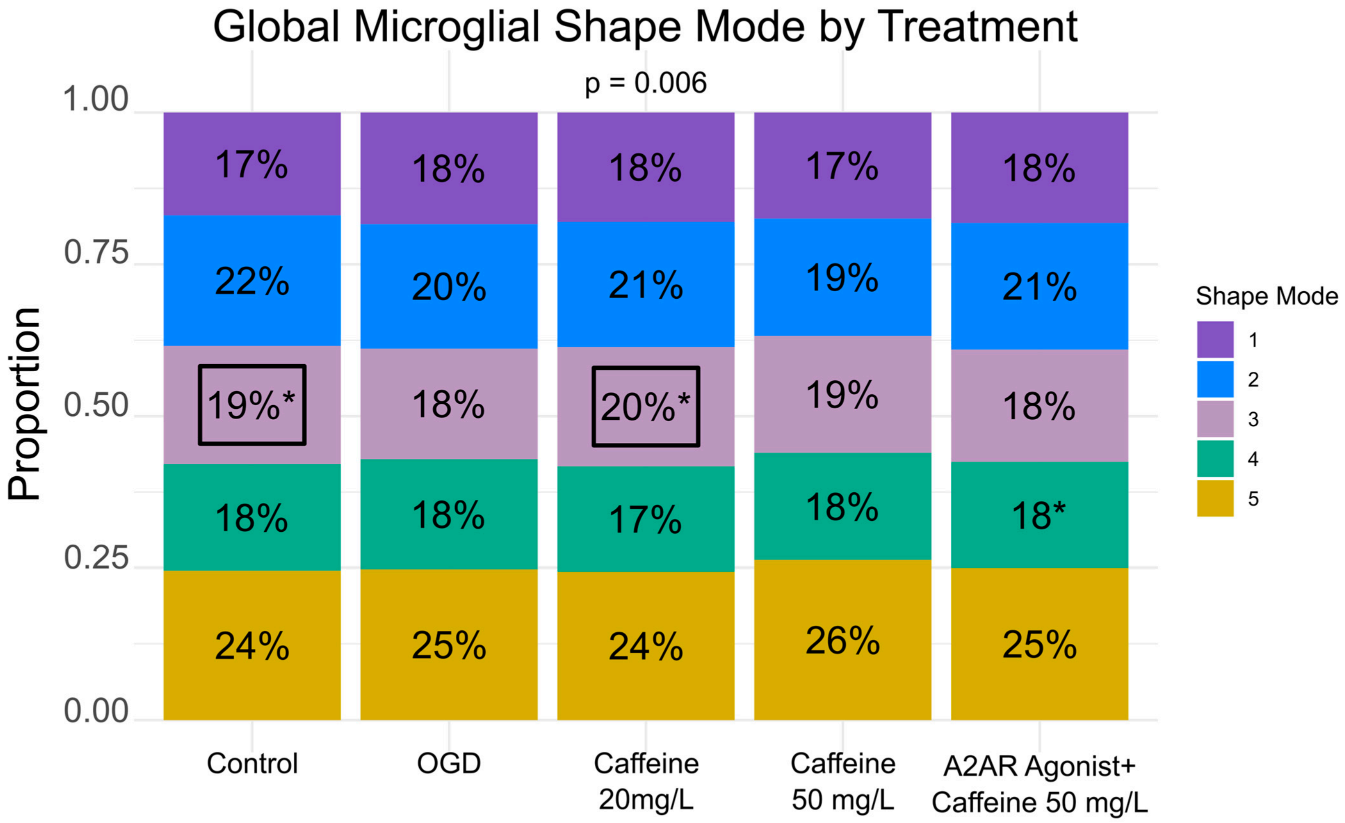

Supplemental Figure S5. Proportion of global microglial shape mode by treatment. Proportion of shape mode 3 was significantly higher in control and caffeine 20 mg/L compared to oxygen-glucose deprivation (OGD). Logistic mixed effect models with fixed effect for region and random effect by slice and experiment were used to assess each shape model individually. Overall, the distribution of shape modes significantly differed between treatment groups after adjusting for region (p=0.006).

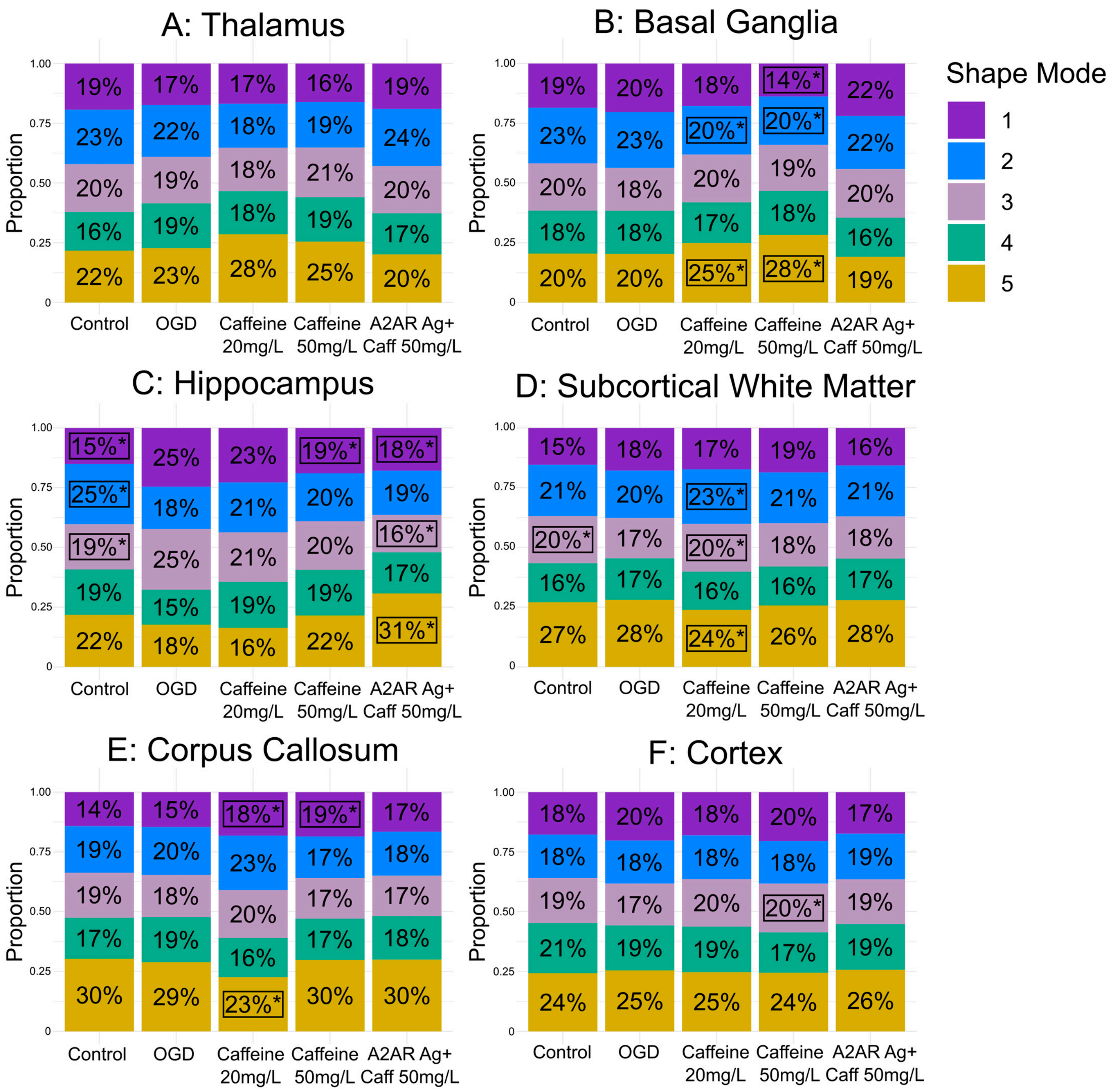

Supplemental Figure S6. Regional analyses of microglial shape modes. No shape mode (SM) differences were observed in the thalamus (**A**). In the basal ganglia, caffeine 20 mg/L reduced SM1 and both doses of caffeine altered SM2/SM5 proportions (**B**). In the hippocampus, caffeine ± A2AR agonist reduced SM1, with additional effects on SM3 and SM5 seen A2AR agonist with caffeine 50 mg/L (**C**). In the subcortical white matter, caffeine 20 mg/L increased SM2/SM3 and decreased SM5 (**D**). In the corpus callosum, both caffeine doses increased SM1, while 20 mg/L decreased SM5 (**E**). In the cortex, SM3 was higher in OGD than caffeine 50 mg/L. SM3 was increased in the cortex by caffeine 50 mg/L compared to OGD (**F**). Significant proportions are bolded with an asterisk.
